# Household Air Pollution Related to Housing Characteristics and Cooking Conditions in Jimma Town, Ethiopia

**DOI:** 10.4314/ejhs.v34i3.2

**Published:** 2024-05

**Authors:** Elias Mulat, Dessalegn Tamiru, Kalkidan Hassen Abate

**Affiliations:** 1 Department of Biomedical Sciences, Institute of Health, Jimma University, Jimma, Ethiopia; 2 Department of Nutrition and Dietetics, Food and Nutrition Research Institute, Jimma University, Jimma, Ethiopia

**Keywords:** Particulate Matters, Indoor Air Pollution, Household Air Pollution, Cooking Practice, Solid Fuel, Ethiopia

## Abstract

**Background:**

Globally, a substantial burden of disease is attributable to environmental risk factors including indoor air pollution. Nearly half of the world's population relies on solid fuel. Almost all (98.8%) residents in Ethiopia are dependent on biomass fuel as their basic source of energy for cooking. Thus, we set out to quantify the concentration of indoor air pollutants and household exposures in different housing characteristics and cooking conditions

**Methods:**

A survey was conducted in 280 randomly selected households in Jimma town, Ethiopia. A real-time concentration of fine particulate matters (PM_2.5_, PM_10_) and pollutants including carbon monoxide (CO), carbon dioxide (CO_2_), and volatile organic compounds (VOC) were measured using Laser PM_2.5_ Meter-5800D/5800E and Aeroqual's TM series 500 portable air quality monitors. Data on housing characteristics, kitchen configuration, and ventilation status were collected using observation checklist.

**Results:**

The median concentrations of pollutants in all measured households were PM_2.5_; 294 µg/m3, PM_10_; 270 µg/m3, CO_2_; 577 mg/m3, CO; 7.9 mg/m3, and VOC; 1077 mg/m3. Households that used solid fuels had significantly higher concentration of PM2.5 (U = 53.0, p < 0.001), PM_10_ (U =63.0, p < 0.001),CO_2_ (U = 3519.50, p < 0.001), and CO (U = 3246.0, p < 0.001) than households that used clean fuel.

**Conclusions:**

All households in this study were exposed to high concentration of indoor air pollutants that exceeded WHO's air quality standard Effective strategy should be put in place to reduce the emission of air pollutants and to set air quality management and improvements policy

## Introduction

Globally, a substantial burden of disease is attributable to environmental risk factors including indoor air pollution (1). In lower-income countries, indoor air pollution is mainly caused by burning solid fuels such as wood, crop residues, animal dung, andcharcoal for cooking, lighting, and heating (2). Recent estimates indicated that nearly half of the world's population relies on solid fuel, while disproportionately the figure surpasses 95% in low-income countries where residents are dependent on biomass fuel for energy (3). According to the Ethiopian Demographic and Health Survey (EDHS) report, about 98.8% of rural and 70.6% of urban dwellings in Ethiopia utilize solid fuel as their basic energy source for cooking (4).

In the indoor environment, respirable particulate matters (PM_25_) and inhalable particulate matters (PM_10_) are the commonest type of pollutant raised from the combustion of solid fuel (5). Furthermore, large amounts of hazardous air pollutants including carbon dioxide (CO_2_), carbon monoxide (CO), hydrocarbons, and other toxic particles also are released from biomass fuel use for heat energy (6-8). Suboptimal household characteristics, poorly ventilated kitchens, and inefficient cooking appliances (6-7) multiply the odds of exposure to these pollutants. Furthermore, the type of biomass fuel used and the amount of time spent in cooking can influence the concentration of particulate matter (8), and the level of exposure to indoor airpollution (1, 9).

Indoor air pollution (IAP) poses a serious health threat, claiming the lives of millions of people every year (10). The World Health Organization (WHO) reported that exposure to household air pollution is attributable to 9 million deaths per annum or 16% of all global deaths (2). The majority of these mortality was linked to the adverse squeal of pollutants including respiratory problems (8), cardiovascular disease (11), and infant mortality (12). Evidence is also mounting on the morbid effect of air pollutants on fetal growth and development, child growth (13), and endocrine and immune function (2, 14). The level of indoor air pollution can be estimated from direct quantitative measures of air pollutants (13) or through proxy measures of cooking methods or housing characteristics (1, 10). Direct measurement of specific pollutants can help to determine the concentration and type of hazardous material linked to specific morbidity and mortality. Furthermore, the objectivity of such measurements can help in monitoring and evaluation of interventions aimed at improving indoor air condition (7). Studies also documented alternative proxy measures of pollutant exposure including the type of fuel used, the ventilation status of the kitchen, and the amount of time spent cooking (1).

Recent estimates indicated that only 20% of the urban dwellers and 1% of the rural population in Ethiopia had access to clean energy (14). Importantly, in such a context, pollutant exposure can be worse due to poor housing ventilation design (15), lack of a separate kitchen, traditional stove use (16), and time spent cooking (17). Though there is a paucity of adequate data, available studies reported PM_2.5_ exposure ranging from 136-737 µg/m3 (18-24); PM_10_ ranging from 591-1357 µg/m3 (22); VOCs 233-1361 mg/m3 (22, 23) and for CO rangingfrom 69-600ppm (20, 21). In this study, we set out to quantify the concentration of indoor air pollutants, fuel use patterns, cooking practices, and housing and kitchen characteristics of the households in Jimma town and its rural vicinity.

## Materials and Methods

**Study setting and design**: A cross-sectional household survey was conducted in Jimma town, Ethiopia. Jimma town is located at a distance of 352 km southwest of the capital city, Addis Ababa. The town's is geographical located at 7°41′ N latitude and 36° 50′ E longitude, and its altitude is 5,740 feet (1,750-2000 meters) above sea level. Jimma town has an estimated population density of 239,430, divided into twelve urban and five semi-urban kebeles (25).

**Study population and sampling techniques**: The study selected households from five kebeles of Jimma City: Kofe, Garuke, Babala, Ginjo Guduru and Awetu Mendera. The selection was part of a cohort study that examined the effects of indoor air pollution on child linear growth. The sample size was calculated using the formula for the estimation of two means using G-Power V.3.1.9.7, Taking type 1 error of 5%, 90% power, a design effect of 1.5, and an effect size of 0.5. After accounting for 10% non-response rate, the study included 280 households.

The study used a three-stage sampling technique to ensure randomization at different levels. First, it randomly selected 30% representative kebeles (five kebeles) from 17 kebeles found in the study area using a lottery method. Second, it randomly selected villages from each of the chosen kebeles. Third, it randomly selected households from the villages based on the combination of fuel use and kitchen configuration, which are the main factors influencing indoor air pollution exposure. The household selection followed the probability proportionate to size criteria.

### Data collection procedures, techniques, and tools

**Sociodemographic, socioeconomic, and housing data**: Information related to study participants' demographics, housing characteristics, kitchen characteristics, and cooking activity patterns were gathered using a questionnaire adapted from a standardized Indoor Air Housing Questionnaire prepared by the International Laboratory for Air Quality and Health WHO collaborating Canter (26). The height and length of the kitchen were also measured using a non-stretchable measuring tape to obtain the kitchen surface area. “Kitchen ventilation was assessed using wall and roof openings in relation to room surface area” (27). Accordingly, aggregate ventilation indicators were computed based on adding the four individual ventilation indicators. Finally, kitchen ventilation was categorized as poor, moderate, and substantial/good. Kitchens are categorized as having poor ventilation in cases of fully closed wall structure, solid roof structure, no opening in the kitchen except for the door, surface area less than 10m^2^, kitchen inside main building, and no opening doors and windows while cooking. Moderate ventilation was defined as wall structure- fully closed, permeable with/without openings; roof structure- permeable/kitchen; openings- small or medium-sized openings/ kitchen surface area - 10 to 15 m^2^ /kitchen separation attached to main building/frequency of opening doors and windows while cooking: rarely). Good/substantial ventilation was considered when the wall structure- semi-enclosed; one to three walls to the roof; Roof structure- permeable with openings; Kitchen openings- significant openings; Kitchen surface area- more than 15 m^2^; Kitchen separationseparated from main building; frequency of opening doors and windows while cookingoften or always (27). The household wealth index was determined using PCA and categorized as lowest, middle, and Highest tertiles (28).

**Exposure assessment/measurement of households air pollutants**: The concentration of Particulate Matters (PM2.5, PM10) and gaseousair pollutants (CO_2_, CO, and VOC), were measured to assess the exposure level of the households to indoor air pollutants. Measurements were performed in the kitchen area during cooking time when the fire was lit for avenge duration of 30 minutes to one hour. The monitoring equipment was positioned at about 1 m above the ground and within 1 m from the cooking stove.

Household PM_2.5_ and PM_10_ concentrations were measured using the Laser PM2.5 Meter-5800D/5800E (detection range 0-999.9 µg/m3) with a minimum particle detection diameter of 0.3µm. The device has an internal laser scattering measuring principle with a relative accuracy of 20% or 15g/m3 MAX. The device is adjusted in the real-time measurement mode with a 1-minute interval time of PM_2.5_ measurement time. The monitors were calibrated to a zero filter before and after each sampling period.

Measurements of indoor air pollutant levels of carbon monoxide (CO), carbon dioxide (CO_2_), and volatile organic compounds (VOC) were performed using Aeroqual's ™ Series 500 portable air quality monitor device. The monitor base is an ergonomically designed electronics platform into which we plug our chosen sensor head(s). Sensors are housed within an interchangeable sensor heads that attaches to the monitor base. Each sensor contains a single gas or particle sensor.

A real-time CO_2_ level was measured using a non-dispersive infrared (NDIR) sensor in the portable monitor range. The detection range for CO_2_ is between 0–2000 ppm with a minimum detection limit of 10 ppm and a resolution of 1 ppm. The accuracy of factory calibration for the sensor is r 10 ppm + 5%. The measurement response time of the device is also 120 seconds. Similarly, in the portable monitor range, we measure carbon monoxide using a gas-sensitive electrochemical (GSE) sensor. The detection range for CO is between 0–100 ppm with a minimum detection limit of 0.2 ppm and a resolution of 0.1 ppm. The accuracy of factory calibration for the sensor is r 1 ppm for 0-10 ppm and 10% for 10-100 ppm.

In the portable monitor range, we also measured VOC using a photo-ionization detector (PID). Like all sensors in the portable monitor, the sensor benefits from active fan sampling and comes factory-calibrated. The sensor has a detection range of 0–2000 PPM and a minimum detection limit of 1 PPM with 1-PPM resolution. The accuracy of factory calibration for the sensor is r 0.2ppm+10% 1000 ppm and 0.1>1000 ppm (29).

**Data management and analysis**: Descriptive statistical results were generated for the outcome and independent variables. In categorical variables, percentages were generated from frequency scores. As central measures of continuous variables, medians with interquartile ranges were used to analyze indoor air pollutant concentrations. Levels of indoor pollutants were compared between households' fuel types used for cooking (solid, mixed, and clean fuel), kitchen characteristics (Indoor Kitchen with Partition, Indoor Kitchen without Partition, and Separate Kitchen outside the House), stove type used for cooking traditional three stone, improved and electric stove). The relationship between the predictor variable and the concentration of household air pollutants was examined by using Kruskal-Wallis test and A Mann-Whitney U test. In addition, Spearman correlation analysis was conducted to evaluate the linear relationship between continuous variables. P-values of <0.05 were considered statistically significant. Statistical Package for Social Science (SPSS) Version 25 software was used for all statistical analysis.

**Ethics**: Ethical clearance was obtained from Jimma University IRB. Written informed consent, with the necessary information in the consent form, was obtained from each participant before the interview.

## Results

**Socio-economic and housing characteristics**: Two-hundred eighty households participated in the study. The mean age of participants was 36.47 (SD: 14.42) years, and nearly half (48.9 %) of them had no formal education. The households had a mean family size of 4.7 (SD: 1.83) and had lived in the house for an average of 9.59 years (SD: 7.2). About a third (32.9%) of the households were in the lowest wealth tertile, while the rest were in the middle (30.7%) or highest (36.4%) tertile. Regarding the materials used for housing construction, Most of the houses had roofs made of corrugated metal sheets (81.9%), walls made of wood (68.9%), and floors made of mud (65%) (Table 1).

**Table 1:** Socio-economic and household characteristics of study participants, Jimma Ethiopia, 2023

Variable/Housing Characteristics	Category	Frequency (%)
Roof Materials	Corrugated Iron	229 (81.9)
	Grass Leaves/ Tach	51(18.2)
Wall material	Wood/ Mud	193 (68.9)
	Burnt Bricks/ Cement Concrete	87 (31.1)
Floor	Clay/Mud	182 (65.0)
	Cement	98 (35.0)
Number of rooms in the house (Mean ± SD)	3.67 ± 1.08	
Numher of doors in the house Mean ± SD	2.00 ± 0 55	
Number of windows in the house (Mean ± SD)	2.18±0.91	
Dwelling years in the house (Mean ± SD)	9.59 ±7.20	
Family size (Mean ± SD)	4.76 ± 1.83	
Household Educational status	No formal education	137 (48.9)
	Primary level education	96 (34.3)
	Secondary level and above	47 (16.8)
Wealth index	Lowest	92(32.9)
	Middle	86 (30.7)
	Highest	102 (36.4)
Pet in the households	Yes	129 (46.1)
	No	151(53.9)

**Household fuel sources and pollutant concentrations**: Solid fuels (wood = 36.4% and crop residues = 13.6%) were the main sources of energy for cooking for 50% of the households in the study, while the other 50% used clean fuel (electricity). The pollutants had the following median concentrations: PM25; 293.95 µg/m3 (IQR: 770.26), PM_10_; 270.85 µg/m3 (IQR: 1893.38), CO_2_; 577.50 mg/m3 (IQR: 350), CO; 7.90 mg/m3 (IQR: 8.20), and VOC; 1077.50 mg/m3 (IQR: 861).

**Indoor air pollutant concentration across fuel types**: Houses that used solid fuel had much higher median levels of PM_2.5_ (905.10µg/m3, IQR: 336.50) and PM_10_(1999.0 µg/m3, IQR: 1827.30) which is significantly higher as compared to houses that used clean fuel, for PM_2._**_5_**,99.00 µg/m3 (IQR: 75.80) and for PM_10_, (119.70 µg/m3, IQR: 73.10) (p < 0.001). The Mann-Whitney U test showed that the difference in the concentrations of PM2.5 and PM10 by fuel types was statistically significant (U = 53.0, Z = −14.436, p < 0.001 for PM2.5 and U =63.0, Z = −14.502, p < 0.001 for PM10) (Table 2).

**Table 2:** Results of Mann Whitney analysis for indoor air pollutants concentration and different fuel types used for cooking

					*Mann Whitnley U test*		
					
Pollutants	Fuel type	N	Median(IQR)	Mean rank	*U*	*Z*	*P* *
PM 2.5	Solid fuel	140	905.10(336.50)	210.12	53.00	−14.436	<.001
µg/m3	Clean fuel	140	99.00 (75.80)	78.88			
PM10 µg/m3	Solid fuel	140	1999(1827.30)	210.05	63.00	−14.502	<.001
	Clean fuel	140	119.70(73.10)	70.95			
CO2 mg/m3	Solid fuel	140	893.00(1186)	185.36	5319.50	−7.273	<.001
	Clean fuel	140	507.00(123)	95.64			
CO mg/m3	Solid fuel	112	11.25(20.75)	118.52	3246.0	−4.445	.002
	Clean fuel	91	7.00(4.60)	81.67			
VOC mg/m3	Solid fuel	140	1550.50(583)	195.67	2076.00	−11.40	<.001
	Clean fuel	140	817(347)	85.33			

PM2.5= Particulate matter< 2.5 µm in diameter. PM10= Particulate matter< 10 µm in diameter. CO2=carbon dioxide CO=carbon monoxide. VOC= Volatile Organic Compound. IQR= Interquartile Range.

* P values refer to the difference between the twofuel types compared. Tested with the Mann–Whitney U test for medians

Similarly, significantly higher levels of CO_2_ were measured in houses that used solid fuel (median 893.00mg/m3, IQR: 1186) compared to houses which used clean fuel (median 507.0, IQR: 123)mg/m3. The same trend was observed in the case of CO: solid fuel (median 11.25mg/m3, IQR: 20.75)mg/m3, and clean fuel (median 7.0, IQR: 4.60)mg/m3 and VOC; solid fuel (median 1550.50, IQR: 583)µg/m3, and clean fuel (median 817.50mg/m3, IQR: 34) (p< 0.001).

The Mann-Whitney U test results also indicated that households that used solid fuels had a significantly higher concentration of CO_2_ (U = 3519.50, Z = −7.273, p < 0.001), CO (U = 3246.0, Z = −4.445, p < 0.001) and VOC (U = 2073.0, Z = −11.40, p < 0.001) than households that used clean fuel (Table 2).

**Kitchen configurations, cooking-related conditions, and level of indoor air pollutants**: The study assessed the kitchen configuration of the households and found that most of them (166 or 59.28%) had a separate kitchen outside the main living room. The rest of the households had an indoor kitchen in the main living room, either with a partition (44 or 15.71%) or without a partition (70 or 25%). The study also examined the kitchen ventilation and found that nearly half of the kitchens (44.64%) had poor ventilation, while the others had moderate (119 or 42.5%) or good (36 or 12.86%) ventilation. Regarding the primary stove types, about 40.4% of the households used traditional three stone stoves for cooking. Most of the households (58.2%) cooked their meals twice a day, in the morning and evening. The average time spent in the kitchen while cooking was 3.34 ± 0.20 hours (Table 3).

**Table 3:** kitchen configurations and cooking-related conditions of the study households, Jimma, Ethiopia

Variables	Category	Frequency(%)
Kitchen configurations	Indoor Kitchen With Partition	44 (15.7)
	Indoor Kitchen Without Partition	70 (25.0)
	Separate Kitchen Outside The House	166 (59.3)
Wall structure	Fully closed-Impermeable	114 (40.7)
	Fully closed-permeable	105 (37.5)
	Semi-enclosed	41(16.4)
Roof Structure	solid roof	136 (48.6)
	permeable without opening	51(18.2)
	permeable with opening	85 (30.4)
Kitchen openings	no opening	104 (37.1)
	small/medium size opening	66 (23.6)
	significant opening	38 (13.6)
Kitchen window	Yes	85 (30.4)
	No	195 (69.6)
Frequency of opening door	Never	209 (74.6)
and window while cooking	Rarely	71(25.4)
	often	
Kitchen space area m^2^	<10m^2^	98 (35.0)
	10-15m^2^	152 (54.3)
	>15m^2^	30 (10.7)
Mean± SD	10.59 ±3.42	
AggregateKitchen ventilation status	Poor	125 (44.6)
	Medium	119 (42.5)
	Good	36 (12.9)
Primary Stove type	Traditional stove made of three stones	113 (40.4)
	Mud improved	27 (9.6)
	Electric stove	140 (50.0)
Daily Cooking frequency	Twice	163 (58.2)
	Three times	117 (41.8)
Daily Time spent for cooking in hour		
Mean± SD	3.34 ±0.20	

A significantly higher median level of PM_2.5_ (915.10 µg/m3) was measured in the kitchen located inside the main living houses compared to the kitchen separated with partition, 129.15 µg/m3, and detached kitchen outside the house, 126.70 µg/m3 (p< 0.001). The highest median level of PM_10_ was also measured in the kitchen located inside the main living houses (median 1999.0 µg/m3 compared to kitchen separated with partition, 136.40 µg/m3 and detached kitchen outside the house, 167.85µg/m3 (p<0.001). The Kruskal-Wallis test based on household kitchen characteristics indicated a significant difference on the concentration of PM_2.5_; χ^2^(2) = 73.419, p < 0.001, PM_10_; χ^2^(2) = 82.774, p < 0.001, CO_2_; χ^2^(2) = 91.036, p < 0.001, CO; χ^2^(2) = 15.990, p < 0.001, and VOC; χ^2^(2) = 63.190, across the three kitchen configurations, p < 0.001( Table 4).

**Table 4:** A Kruskal-Wallis test based on Households kitchen configuration and concentration of indoor air pollutants, Jimma, Ethiopia

Ranks						
			
Pollutants	kitchen configuration	N (280)	Mean Rank	Median(IQR)	*X^2^(df=2)*	*p*
PM2.5	Indoor With Partition	44	142.77	129.15(623.38)	93.913	< 0.001
µg/m3	Indoor Without Partition	70	214.21	915.20(334.38)		
	Separate Outside the House	166	108.82	126.70(510.33)		
PM10	Indoor With Partition	44	140.45	136.40(823.63)	92.389	< 0.001
µg/m3	Indoor Without Partition	70	217.09	1999.0(829.75)		
	Separate Outside the House	166	108.21	167.85(529.85)		
CO2 mg/m3	Indoor With Partition	44	121.07	541.00(155)	67.040	< 0.001
	Indoor Without Partition	70	224.47	1778.50(881)		
	Separate Outside the House	166	110.24	538.50(1626)		
CO mg/ m3	Indoor With Partition	31	93.92	6.05(6.15)	8.603	0.014
	Indoor Without Partition	56	129.33	12.50(22.38)		
	Separate Outside the House	116	90.97	7.30(6.60)		
VOC mg/m3	Indoor With Partition	44	145.69	952.50(865)	70.929	< 0.001
	Indoor Without Partition	70	207.12	1622,0(707)		
	Separate Outside the House	166	111.03	894.50(641)		

PM2.5= Particulate Matter < 2.5 µm in diameter. PM10= Particulate Matter < 10 µm in diameter.CO2=carbon dioxide CO=carbon monoxide. VOC= Volatile Organic Compound. IQR= Inter Quartile Range. X^2^= chi-squared (test statistics, df = degree of freedom, 2. *P-values refer to the difference between the three kitchen types compared at a 0.05 significance level. Tested with the Kruskal-Walis test for medians.

Kruskal-Wallis test was also conducted to determine the effect of the status of kitchen ventilation (poor, moderate, and good) on the median concentration of household air pollutants. The result indicated a statistically significant difference in concentration of PM_2.5_; χ^2^(2) = 167.930, p < 0.001, PM_10_; χ^2^(2) = 143.540, p < 0.001, CO_2_; χ^2^(2) = 51.050, p < 0.001, CO; χ^2^(2) = 15.100, p = 0.001, and VOC; χ^2^(2) = 66.734, p < 0.001. Further, a pairwise test also revealed that households with poor kitchen ventilation status had a significantly higher concentration of indoor air pollutants compared to households with moderate and good kitchen ventilation status (Table 5).

**Table 5:** Kruskal-Wallis test based on kitchen ventilation status and concentration of indoor air pollutants, Jimma, Ethiopia

Ranks				Pairwise Comparisons			

Pollutants	kitchenventilation	Mean Rank	Median(IQR)	samplel-sample2	*X^2^(df=2)*	*SE*	*p*
PM2.5	Poor(n=125)	208.13	912.20(326)	Good-moderate	46.872	3.054	0.002
µg/m3	Moderate (n=119)	96.84	114.40(175)	Good-poor	158.160	10.362	< 0.001
	Good (n=36)	49.97	48.80(63.40)	Moderate-poor	111.287	10.768	< 0.001
PM10	Poor(n=125)	1101.15	1999(1101.15)	Good-moderate	40.249	15.265	0.002
µg/m3	Moderate (119)	152.10	139.10(152.10)	Good-poor	143.652	15.180	< 0.001
	Good (36)	84.70	86.55(84.70)	Moderate-poor	103.403	10.278	< 0.001
CO2	Poor(n=125)	1248	847(1248)	Good-moderate	6.940	15.397	0.652
mg/m3	Moderate (119)	159	538(159)	Good-poor	74.718	15.331	0.029
	Good (36)	137	532(137)	Moderate-poor	67.778	10.367	< 0.001
CO mg/	Poor(n=125)	21.60	10.60(21.60)	Good-moderate	9.528	13.860	0.492
m3	Moderate (119)	6.38	7.0(6.38)	Good-poor	38.995	13.610	0.004
	Good (36)	5.60	7.0(5.60)	Moderate-poor	29.468	8.791	0.001
VOC	Poor(n=125)	183.64	1488(705)	Good-moderate	24.944	15.402	0.105
mg/m3	Moderate (119)	11.50	893(485)	Good-poor	97.088	15.315	< 0.001
	Good (36)	86.56	747(459)	Moderate-poor	72.144	10.370	< 0.001

PM2.5= Particulate Matter < 2.5 µm in diameter. PM10= Particulate Matter < 10 µm in diameter.CO2=carbon dioxide CO=carbon monoxide. VOC= Volatile Organic Compound. IQR= Inter Quartile Range. X^2^= chi-squared (test statistics, df = degree of freedom, 2. SE = standard error. *P-values refer to the difference between the three ventilation statuses compared at a 0.05 significance level. Tested with the Kruskal-Walis test for medians.

**Concentration of Indoor air pollutants across primary stove types used for cooking**: The median level of PM_2.5_ µg/m3 in houses using traditional three-stone stoves was 950.80µg/m3(IQR: 253.15) which is significantly higher compared to houses that used improved stoves, 560.60 µg/m3(IQR: 288.50), and electric stoves, 94.42µg/m3 (IQR: 69.48). The highest median level of PM_10_µg/m^3^ was measured in houses using traditional three-stone stoves (median 1999.0, IQR: 1013.20) in the houses using improved stoves (median 930.40, IQR: 1449)µg/m3 and electric stove users (median 106.10, IQR: 74.93). Similarly, significantly higher levels of CO_2_ mg/m^3^ were measured in houses using traditional three-stone stoves (median 847, IQR, 1240) compared to houses using improved stoves (median 707, IQR: 871) and electric stoves (median 513, IQR: 144). The same pattern was observed in the case of CO mg/m^3^: traditional three-stone stove (median 10.60, IQR: 16.60), improved stove (median 9.70, IQR: 13.70) and electric stove (median 7.0, IQR: 4.60) and VOC µg/m^3^; traditional three stone stove (median 1518, IQR: 615), improved stove (median 1519, IQR: 604), and electric stove (median 722, IQR:352).

Kruskal-Wallis test was conducted to examine the effect of the three different stoves (traditional three-stone, improved, and electric stoves) on the median concentration of indoor air pollutants. The results revealed that traditional three-stone stove use resulted in a significantly higher concentration of PM_2.5_; χ^2^ (2) = 214.575, p < 0.001, PM; PM_10_; µ^2^ (2) = 212.629, p < 0.001, CO_2_; χ^2^ (2) = 85.999, p < 0.001, CO; χ^2^ (2) = 19.786, p = 0.004, VOC; χ^2^ (2) = 130.059, p < 0.001 (Table 6).

**Table 6:** Kruskal-Wallis test based on primary stove type and concentration of indoor air pollutants, Jimma, Ethiopia

Ranks			Pairwise Comparisons			

Pollutants	Stove types	Mean Rank	samplel-sample2	*X^2^(df=2)*	*SE*	*p*
PM2.5	Traditional	218.40	Electric-Traditional	117.920	16.962	< 0.001*
µg/m3	Mud-Improved	175.48	Electric-Improved	144.161	10.205	< 0.001*
	Electric	69.48	Traditional-Improved	26.240	17.286	0.013*
PM10	Traditional	215.11	Electric-Traditional	117.920	16.868	< 0.001*
µg/m3	Mud-Improved	188.87	Electric-Improved	144.161	10.149	< 0.001*
	Electric	70.95	Traditional-Improved	26.240	17.191	0.127
CO2 mg/m3	Traditional	185.23	Electric-Traditional	89.586	10.237	< 0.001*
	Mud-Improved	185.93	Electric-Improved	90.287	17.014	< 0.001*
	Electric	95.64	Traditional-Improved	−.700	17.340	0.968
CO mg/ m3	Traditional	118.02	Electric-Traditional	36.347	8.782	< 0.001*
	Mud-Improved	120.35	Electric-Improved	38.684	13.479	0.004*
	Electric	81.67	Traditional-Improved	−2.337	13.527	0.863
VOC mg/m3	Traditional	194.86	Electric-Traditional	109.530	10.240	< 0.001*
	Mud-Improved	199.07	Electric-Improved	113.746	17.019	< 0.001*
	Electric	85.33	Traditional-Improved	−4.216	17.345	0.808

PM2.5= Particulate Matter < 2.5 µm in diameter. PM10= Particulate Matter < 10 µm in diameter.CO2=carbon dioxide CO=carbon monoxide. VOC= Volatile Organic Compound. X^2^= chi-squared (test statistics, df = degree of freedom, 2. SE = standard error.

* P-values refer to the difference between the three-stove types compared at 0.05 significance level. Tested with the Kruskal-Walis test for medians.

## Discussion

The present study measured the concentration of indoor air pollutants in different households in Jimma town and peri-urban areas, and it compared how they varied by housing, kitchen, and cooking-related factors. Our study found high levels of household particulate matter (PM2.5=455.37 µg/m3, and PM10 = 819.06 µg/m3), which was more than 18 times higher than the WHO recommended values of 25 and 45 g/m3 for PM2.5 and PM10, respectively (2, 30). This finding corroborates the findings of several studies conducted in similar settings in Ethiopia (20-22), Ghana (31), and India (32).

Ideally, there is no theoretically safe level of exposure for PM_10_ and PM_2.5_, (33); even at relatively low concentrations, small particles with a diameter of less than 10 microns can penetrate deeply into our lungs and enter our bloodstream, harming every major organ (34). Exposure to such pollutants has been linked to a number of human health risks including cardiovascular (11), respiratory diseases, and cancer (7). Furthermore, pregnant women and their developing fetuses are particularly vulnerable to the adverse health effects of air pollution (35), resulting in increased risk of low birth weight, stillbirths, and infant mortality (36).

Furthermore, exceeding the WHO recommended concentration limits, the mean concentrations of CO_2_, CO, and VOC in the present study were 787.30, 12.0, and, 1154.0 mg/m3, respectively (32). Likewise, studies from similar contexts (20, 22, 24), and LMIC settings (31, 38) also reported higher household concentrations of the aforementioned pollutants that exceeded recommended exposure limits. These pollutants particularly, Carbon monoxide (CO) is a deadly gas that, once breathed in, displaces oxygen from the hemoglobin molecule, resulting in the formation of carboxyhemoglobin in our blood, which can cause severe disability or even death. Breathing in high-CO air reduces the amount of oxygen that can be transported in the bloodstream to vital organs such as the heart and brain. It can cause dizziness, headaches, confusion, unconsciousness, impaired vision and coordination, and even death at very high levels.

In the current study, solid fuel user households had considerably high mean PM_10_ and PM_2.5_ CO_2_, CO, and VOC concentrations compared to clean fuel users. These findings were in line with results of other studies in low income countries (19, 34, 37). The type of fuel use and exposure to pollutants remain at par, justifying the need for promoting clean energy at the household level. Many studies indicated that, compared to separate kitchens, there exist higher odds of pollutant exposure with indoor kitchens establishments (18, 27). Furthermore, one of the most significant factors affecting household exposure to toxic air pollutants is the layout of the kitchen and the state of the ventilation system (27). In poorly ventilated kitchens, exposure to indoor air pollutants exceeds the acceptable concentration limit set by WHO (30).

Apart from kitchen characteristics and ventilation, stove types are determinants for pollutant exposure. In this study, households that used a traditional three-stone stove for cooking had higher concentrations of PM 2.5, PM10, CO2, CO, and VOC as compared to improved stove and electric stove users (p <0.01). In line with our findings, previous studies indicated that improved stoves had lower emissions of particulate matter as compared to traditional three-stone stoves (17, 18). Similarly, a comparative analysis of indoor air pollutant concentration between traditional cooking stoves and improved stoves conducted by several researchers (39), revealed a high level of personal exposure to pollutants in traditional cook stoves compared to improved cook stoves.Traditional three-stone stoves lead to incomplete combustion of solid fuel as a result contribute to the emission of a huge amount of health-damaging toxic byproducts.

The issues of indoor air pollution and household air quality is directly line up with United Nations Sustainable Development Goals particularly (40), SDG 3 (substantial reduction of health impacts from hazardous substances), SDG 7 (ensure access to clean energy in homes), and SDG 11 (reduce the environmental impact of cities by improving air quality). It is also linked to SDG 5 (gender equality), as women in developing countries are disproportionately exposed to emissions from burning solid fuels for cooking, thereby subjecting them to a higher risk of indoor air pollution-related diseases (40).

Solid fuels are still in widespread use in the setting and will remain the principal cooking fuel for a large majority of rural households for the foreseeable future. Hence, in this study, it is pinpointed that an effective indoor air pollution mitigation strategy should employ a variety of options including effective promotion and dissemination of improved cooking stoves, promotion of clean energy use, ensuring improved kitchen configuration and ventilation conditions as well as focus on renewable energy sources. Furthermore, an effective strategy should be implemented to lower the emission of air pollutants to the WHO recommended levels, and air quality management and improvement policy should be set.

## References

[1] Bruce N, Pope D, Rehfuess E, Balakrishnan K, Adair-Rohani H, Dora C (2015). WHO indoor air quality guidelines on household fuel combustion: Strategy implications of new evidence on interventions and exposure–risk functions. Atmospheric Environment.

[2] WHO (2014). Burden of disease from household air pollution for 2012.

[3] Shaddick G, Salter JM, Peuch V-H, Ruggeri G, Thomas ML, Mudu P (2020). Global Air quality: an inter-disciplinary approach to exposure assessment for burden of disease analyses. Atmosphere.

[4] Csa I. Central Statistical Agency (CSA) [Ethiopia] and ICF (2016). Ethiopia Demographic and Health Survey, Addis Ababa.

[5] Avakian MD, Dellinger B, Fiedler H, Gullet B, Koshland C, Marklund S (2002). The origin, fate, and health effects of combustion by-products: a research framework. Environmental health perspectives.

[6] Mitra P, Chakraborty D, Mondai NK (2022). Assessment of household air pollution exposure of tribal women. Science of the Total Environment.

[7] Nandre CD, Kulkarni SS (2018). Trends of indoor air pollution and respiratory tract infections on global scale: A review. Asian Man (The)-An International Journal.

[8] Smith KR (2002). Indoor air pollution in developing countries: recommendations for research. Indoor air.

[9] Faris K (2002). Survey of indoor air pollution problems in the rural communities of Jimma, Southwest Ethiopia. Ethiopian Journal of Health Sciences.

[10] Tran VV, Park D, Lee Y-C (2020). Indoor air pollution, related human diseases, and recent trends in the control and improvement of indoor air quality. International journal of environmental research and public health.

[11] Hadley MB, Baumgartner J, Vedanthan R (2018). Developing a clinical approach to air pollution and cardiovascular health. Circulation.

[12] Lu C, Zhang W, Zheng X, Sun J, Chen L, Deng Q (2020). Combined effects of ambient air pollution and home environmental factors on low birth weight. Chemosphere.

[13] Smith KR, Bruce N, Balakrishnan K, Adair-Rohani H, Balmes J, Chafe Z (2014). Millions dead: how do we know and what does it mean? Methods used in the comparative risk assessment of household air pollution. Annu Rev Public Health.

[14] Leech JA, Nelson WC, Burnett RT, Aaron S, Raizenne ME (2002). It's about time: a comparison of Canadian and American time–activity patterns. Journal of Exposure Science & Environmental Epidemiology.

[15] Guta DD (2018). Determinants of household adoption of solar energy technology in rural Ethiopia. Journal of Cleaner Production.

[16] Fullerton DG, Bruce N, Gordon SB (2008). Indoor air pollution from biomass fuel smoke is a major health concern in the developing world. Transactions of the Royal Society of Tropical Medicine and Hygiene.

[17] Adane MM, Alene GD, Mereta ST (2021). Biomass-fuelled improved cookstove intervention to prevent household air pollution in Northwest Ethiopia: a cluster randomized controlled trial. Environmental health and preventive medicine.

[18] Tamire M, Kumie A, Addissie A, Ayalew M, Boman J, Skovbjerg S (2021). High Levels of Fine Particulate Matter (PM2. 5) Concentrations from Burning Solid Fuels in Rural Households of Butajira, Ethiopia. International Journal of Environmental Research and Public Health.

[19] Admasie A, Kumie A, Worku A, Tsehayu W (2019). Household fine particulate matter (PM2. 5) concentrations from cooking fuels: the case in an urban setting, Wolaita Sodo, Ethiopia. Air Quality, Atmosphere & Health.

[20] Graham M (2011). Mixed Methods Approach to Assessing Indoor Air Pollution Among Women in Addis Ababa, Ethiopia.

[21] Sanbata H, Asfaw A, Kumie A (2014). Indoor air pollution in slum neighbourhoods of Addis Ababa, Ethiopia. Atmospheric Environment.

[22] Embiale A, Chandravanshi BS, Zewge F, Sahle-Demessie E (2022). Health risk assessment of total volatile organic compounds, particulate matters and trace elements in PM10 in typical living rooms in Addis Ababa, Ethiopia. International Journal of Environmental Analytical Chemistry.

[23] Asefa EM, Mergia MT (2022). Human exposure to indoor air pollution in Ethiopian households. Heliyon.

[24] Weldetinsae A, Usinger J (2008). Indoor Air Pollution in Rural Tigray. GNU Free Documentation License.

[25] Agency CS (2010). The 2007 population and housing census of Ethiopia, result for Oromia region.

[26] American Thoracic S (1978). Recommended respiratory disease questionnaires for use with adults and children in epidemiological research. Am Rev Respir Dis.

[27] Lenz L, Bensch G, Chartier R, Kane M, Ankel-Peters J, Jeuland M (2023). Releasing the killer from the kitchen? Ventilation and air pollution from biomass cooking. Development Engineering.

[28] Demographic E (2017). Health Survey, 2016: ICF International.

[29] Lin C, Gillespie J, Schuder MD, Duberstein W, Beverland IJ, Heal MR (2015). Evaluation and calibration of Aeroqual series 500 portable gas sensors for accurate measurement of ambient ozone and nitrogen dioxide. Atmospheric Environment.

[30] World Health O (2021). WHO global air quality guidelines: particulate matter (PM2. 5 and PM10), ozone, nitrogen dioxide, sulfur dioxide and carbon monoxide.

[31] Van Vliet EDS, Asante K, Jack DW, Kinney PL, Whyatt RM, Chillrud SN (2013). Personal exposures to fine particulate matter and black carbon in households cooking with biomass fuels in rural Ghana. Environmental research.

[32] Balakrishnan K, Ghosh S, Ganguli B, Sambandam S, Bruce N, Barnes DF (2013). State and national household concentrations of PM2. 5 from solid cookfuel use: results from measurements and modeling in India for estimation of the global burden of disease. Environmental Health.

[33] Bové H, Bongaerts E, Slenders E, Bijnens EM, Saenen ND, Gyselaers W (2019). Ambient black carbon particles reach the fetal side of human placenta. Nature communications.

[34] Liu NM, Miyashita L, Mäher BA, McPhail G, Jones CJ, Barratt B (2021). Evidence for the presence of air pollution nanoparticles in placental tissue cells. Science of The Total Environment.

[35] Wylie BJ, Matechi E, Kishashu Y, Fawzi W, Premji Z, Coull BA (2017). Placental pathology associated with household air pollution in a cohort of pregnant women from Dar es Salaam, Tanzania. Environmental health perspectives.

[36] Madhloum N, Nawrot TS, Gyselaers W, Roels HA, Bijnens E, Vanpoucke C (2019). Neonatal blood pressure in association with prenatal air pollution exposure, traffic, and land use indicators: An ENVIRONAGE birth cohort study. Environment international.

[37] (2015). Depressed height gain of children associated with intrauterine exposure to polycyclic aromatic hydrocarbons (PAH) and heavy metals: the cohort prospective study. Environmental research.

[38] Shrestha IL, Shrestha SL (2005). Indoor air pollution from biomass fuels and respiratory health of the exposed population in Nepalese households. International journal of occupational and environmental health.

[39] Singh A, Tuladhar B, Bajracharya K, Pillarisetti A (2012). Assessment of effectiveness of improved cook stoves in reducing indoor air pollution and improving health in Nepal. Energy for sustainable development.

[40] Zhang J, Smith KR (2003). Indoor air pollution: a global health concern. British medical bulletin.

